# Reports of Pennellidae Burmeister, 1835 (Copepoda: Siphonostomatoida) post metamorphosed females off the coast of southern Africa

**DOI:** 10.1007/s11230-024-10157-0

**Published:** 2024-04-22

**Authors:** Makwena M. Sebone, Susan M. Dippenaar

**Affiliations:** Department of Biodiversity, University of Limpopo, Private Bag X1106, Sovenga, 0727 South Africa

## Abstract

Seven species belonging to Pennellidae are reported from marine teleosts caught off southern Africa. Additionally, complete re-descriptions are provided for *Propeniculus stromatei* and *Sarcotretes scopeli*. Examination of *Lernaeenicus gonostomae*, deposited in the Iziko South African Museum, indicated that it has the morphological features of *Sarcotretes* rather than *Lernaeenicus* and thus should be moved to *Sarcotretes* i.e. *S*. *gonostomae* **n. comb.** for which a re-description is also provided. Reports of new host records include those of *Pennella instructa* from *Seriola lalandi*; *Propeniculus stromatei* from *Rhabdosargus holubi* and *Pomadasys commersonnii*; *Sarcotretes scopeli* from *Nansenia tenera*, and *Sarcotretes longirostris* from *Centrolophus niger*. New geographical records include those of *P*. *instructa*, *P*. *stromatei*, *S*. *scopeli*, *S*. *longirostris*, and *L*. *longiventris* off southern Africa. Additionally, an attempt to estimate the evolutionary relationships amongst some genera is done from partial COI sequences deposited in Genbank.

## Introduction

Pennellidae (Copepoda: Siphonostomatoida) consists of 25 valid genera and 148 species (Walter & Boxshall, 2023; Yumura et al., 2024) symbiotic on marine fish and mammals (Kabata, 1979; Boxshall & Halsey, 2004). Adult metamorphosed female pennellids have large bodies and lose their maxillipeds as mesoparasite adaptations (Boxshall & Halsey, 2004). These metamorphosed females exhibit variable morphology regarding for example, the structure of the cephalothorax (ranging from a simple cephalothorax to development of a cephalic holdfast organ), the trunk (varying from straight trunks to sigmoid trunks), the abdomen (varying from an indistinct abdomen to an abdomen embedded with posterior processes) and the egg strings (varying from straight egg strings to curled egg strings) (Castro-Romero, 2014).

This family has a history of intergeneric misidentifications (Kabata, 1979; Castro-Romero, 2014; Walter & Boxshall, 2023). For example, revised species of *Peniculus* von Nordmann, 1832 were transferred to several genera including *Peniculisa* Wilson C.B., 1917; *Metapeniculus* Castro-Romero & Baeza-Kuroki, 1985; *Propeniculus* Castro-Romero, 2014, and *Pseudopeniculus* Castro-Romero, 2014 (Wilson, 1917; Castro-Romero & Baeza-Kuroki, 1985; Castro-Romero, 2014). Similarly, several revised species of *Lernaeenicus* Lesueur, 1824 have already been transferred to different genera (e.g. *Sarcotretes* Jungersen, 1911; *Protosarcotretes* Ohtsuka, Lindsay & Izawa, 2018, and *Cardiodectes* Wilson, C.B., 1917) due to possession of morphological characters similar to those of other genera (Wilson, 1917; Ohtsuka et al., 2018).

To date, only six genera and eight species [*Pennella balaenoptera* Koren & Danielssen, 1877 from *Balaenoptera physalus* (Linnaeus); *Pennella filosa* (Linnaeus, 1758) from *Istiompax indica* (Cuvier), *Mola mola* (Linnaeus), *Thunnus albacares* (Bonnaterre), and *Balaenoptera acutorostrata* Lacépède; *Peniculus fistula fistula* von Nordmann, 1832 from *Kaperangus microlepis* (Norman); *Peniculisa furcata* (Krøyer, 1863) from *Paramonacanthus frenatus* (Peters); *Peroderma cylindricum* Heller, 1865 from *Sardinella maderensis* (Lowe); *Cardiodectes bellottii* (Richiardi, 1882) from *Lampanyctodes hectoris* (Günther) and *Gonichthys cocco* (Cocco); *Lernaeenicus gonostomae* Kensley & Grindley, 1973 from *Sigmops elongatus* (Günther); and *Lernaeenicus kabatai* Oldewage, 1989 from *Carangoides equula* (Temminck & Schlegel)] were reported from marine fish and mammals off southern Africa (Barnard, 1955; Perkins, 1983; Dippenaar, 2004).

This paper reports on the Pennellidae species collected from marine fish off Southern Africa, with re-descriptions of *Sarcotretes scopeli* Jungersen, 1911 and *Propeniculus stromatei* (Gnanamuthu, 1951). Notes are provided about the genus *Lernaeenicus*, with synonymizing and re-description of *Lernaeenicus gonostomae* Kensley & Grindley, 1973 based on voucher specimens from the Iziko South African museum. Additionally, the phylogenetic relationships between selected Pennellidae genera, using available mitochondrial COI (cytochrome oxidase I) sequences, are investigated.

## Materials and Methods

Specimens collected from fish caught off the southern African coasts, from 1993 to 2019 were preserved in 70% ethanol. For morphological studies, specimens were stained with a mixture of lactic acid and a small amount of lignin pink, then examined using stereo- and compound microscopes. Some appendages were dissected and drawings were made with the aid of drawing tubes. All measurements were done using a 2 mm stage micrometer and are given as mean (range) mm. Terminology used in morphological descriptions conforms to that of Kabata (1979). Host species names were validated using Froese and Pauly (2023). Voucher specimens for *Pennella instructa*, *Sarcotretes scopeli*, *S*. *longirostris* and *Propeniculus stromatei* were deposited in the Iziko South African museum.

For molecular studies, DNA was extracted from *Sarcotretes scopeli*. Specimens were cut into small pieces and dried in 1.5 microcentrifuge tubes on a heat block at 56°C for approximately 2 hours. Genomic DNA was extracted using the Zymo Research Quick-DNA™ miniprep plus kit, following the protocol for solid tissues. Polymerase Chain Reaction (PCR) was performed to amplify a fragment of the partial mitochondrial (mtDNA) COI gene with the forward primer mICOIintF (GGWACWGGWTGAACWGTWTAYCCYCC) and reverse primer jgHCO2198 (TAIACYTCIGGRTGICCRAARAAYCA) (Geller et al., 2013; Leray et al., 2013) in the Eppendorf Mastercycler. A 25 µl PCR reaction mixture was prepared for each sample, consisting of 12 µl of OneTaq Quick-Load 2X Master Mix with Standard Buffer, 2 µl of each primer, 1-3 µl of template DNA, and double distilled water to adjust the volume. PCR cycling conditions included 94°C (3 min) initial denaturation, followed by 35 cycles of 94°C (30 sec) denaturation, 50°C (1 min) annealing and 68°C (1 min) extension, followed by 68°C (7 min) extension. Purification of PCR products was performed using exoSAP mix and incubated in Eppendorf Mastercycler at 37°C (15 min) and 80°C (15 min) for enzyme inactivation. Sanger sequencing of the purified PCR products was conducted using the Applied Biosystems™ 3730xl DNA Analyzer.

The sequences obtained from the output chromatograms were assembled using CLC genomics workbench 7 (QIAGEN) and carefully examined for nucleotide ambiguities. Forty nine pennellid COI sequences including the genera *Haemobaphes* Steenstrup & Lütken, 1861*, Lernaeenicus*, *Lernaeocera* Blainville, 1822*, Metapeniculus*, *Peniculus, Pennella*, *Propeniculus* and *Trifur* Wilson C.B., 1917 were downloaded from Genbank (Table 1) and compiled into a dataset with one additional generated sequence of *S*. *scopeli* (Genbank accession nr OR807153 - 365bp) with representatives of Caligidae Burmeister, 1835 (*Caligus curtus* Müller O.F., 1785 and *C*. *elongates* von Nordmann, 1832) as outgroup taxa according to Yumura et al. (2022) and Osuna-Cabanillas et al. (2023). Sequences were aligned with Clustal X 2.1 (Thompson et al., 1997) and converted to amino acids using MacClade (Maddison & Maddison, 2001). Furthermore, the dataset was checked for the best model of evolution for Bayesian analysis and Maximum likelihood using jModeltest (Darriba et al., 2012) [AIC = GTR+I+G (-InL = 6398.59; AIC = 13029.18)]. Maximum likelihood was performed using IQtree (Nguyen et al., 2015), with 100000 bootstrap replicates (annotated nodes with bootstrap support of >60%), while Bayesian analysis was performed using MrBayes v3.2 (Ronquist et al., 2012) (nst=6 rates=invgamma), with posterior probabilities percentage of >60% annotated on nodes. The final trees were visualized and edited using Fig tree v1.43 software (Rambaut, 2016).

**Table 1:** Pennellidae species used in the phylogenetic analysis with accession numbers from Genbank.

Accession number	Species name
KT209407.1	*Caligus curtus*
KT209233.1	*Caligus curtus*
KT209299.1	*Caligus elongatus*
KT209384.1	*Caligus elongatus*
KM102172.1	*Lernaeenicus hemirhamphi*
KM102173.1	*Lernaeenicus hemirhamphi*
KM102174.1	*Lernaeenicus hemirhamphi*
KM102165.1	*Lernaeenicus seeri*
KM102164.1	*Lernaeenicus alatus*
KT209575.1	*Lernaeenicus sprattae*
KT209102.1	*Lernaeenicus sprattae*
KT209576.1	*Lernaeenicus sprattae*
MH235914.1	*Lernaeenicus radiatus*
MN520483.1	*Lernaeenicus radiatus*
MH235827.1	*Lernaeenicus radiatus*
KR049061.1	*Haemobaphes pannosus*
KR049062.1	*Haemobaphes pannosus*
LC179654.1	*Haemobaphes diceraus*
LC179655.1	*Haemobaphes diceraus*
LC179653.1	*Haemobaphes diceraus*
MN520484.1	*Lernaeocera branchialis*
MN520485.1	*Lernaeocera branchialis*
MH885291.1	*Pennellidae* sp.
MH885290.1	*Pennellidae* sp.
MH885289.1	*Peniculus* sp.
KU557439.1	*Peniculus* cf*. fistula*
KU557438.1	*Peniculus* cf.* fistula*
MG701292.1	*Pennella balaenoptera*
MG701288.1	*Pennella balaenoptera*
MG701293.1	*Pennella balaenoptera*
MG701289.1	*Pennella balaenoptera*
MG701287.1	*Pennella balaenoptera*
MG701290.1	*Pennella balaenoptera*
MG701291.1	*Pennella balaenoptera*
MG701286.1	*Pennella filosa*
MG701285.1	*Pennella filosa*
MG701282.1	*Pennella filosa*
MG701283.1	*Pennella filosa*
MG701284.1	*Pennella filosa*
KU557415.1	*Metapeniculus antofagastensis*
KU557414.1	*Metapeniculus antofagastensis*
KU557413.1	*Metapeniculus antofagastensis*
KU557416.1	*Metapeniculus antofagastensis*
MH885297.1	*Trifur* sp.
MH885295.1	*Trifur* sp.
MH885296.1	*Trifur* sp.
MH885294.1	*Trifur* sp.
MH885293.1	*Trifur* sp.
MH885292.1	*Trifur* sp.
MH885305.1	*Trifur* sp.
MH885304.1	*Trifur* sp.
MH885306.1	*Trifur* sp.
OP425702.1	*Propeniculus scomberi*
OR807153	*Sarcotretes scopeli*

## Results and discussion

***Pennella*** **Oken, 1815**

*Pennella instructa* Wilson C.B., 1917

Host: *Seriola lalandi* Valenciennes (Carangiformes: Carangidae)

Locality: West coast off South Africa

Material collected and examined: 1♀

### Re-description:

**Fig. 1:**
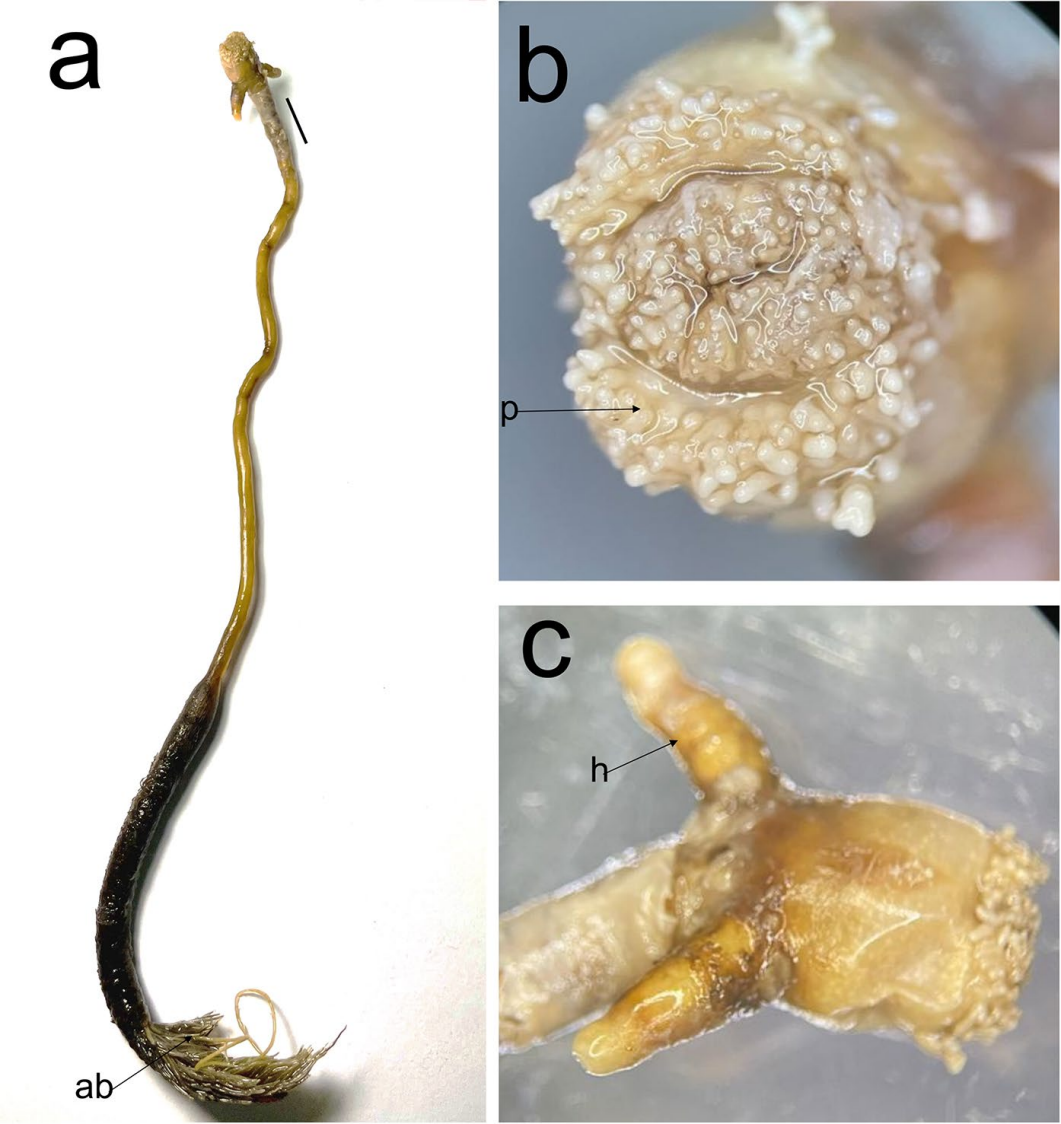
*Pennella instructa* Wilson C.B., 1917, post metamorphosed female; a. habitus, ventral view; b. cephalothorax papillae, anterior view; c. cephalothorax, dorsolateral view. Scale bars: a = 1 mm. (ab = abdomen; p = papillae; h = holdfast horn)

Post metamorphosed females [based on one specimen, fig. 1]. Body length from tip of cephalothorax to tip of abdomen 205 mm, cephalothorax length 8 mm, width 6 mm; cephalothorax horn length 7 mm, width 2 mm; neck-like region length 113 mm, width 2 mm; trunk length 58 mm, width 5 mm; abdomen length 26 mm, width 2.7 mm. Anterior part of cephalothorax (figs. 1a, b) with papillae (p) arranged in four parentheses-like rows, cephalothorax subspherical (figs. 1a, c). Simple cephalic holdfast with two lateral horns (h) (figs. 1a, c), posterolaterally on cephalothorax. Neck-like region elongated, expanding into straight, cylindrical trunk, with elongated abdomen (ab) (fig. 1a) bearing rows of plumules laterally and elongated egg strings, with uniseriate eggs.

### Remarks:

*Pennella* has 10 valid species (Walter & Boxshall, 2023), known to infect a wide range of marine hosts, from teleosts to mammals. Metamorphosed females of this genus vary in total length from large (>100 mm), medium (between 50–100 mm) to small (<50 mm) (Hogans, 2017a). They have cephalothoraces with papillae of different arrangements anteriorly, simple cephalic holdfast with two posterolateral holdfast processes, sometimes with a dorsal holdfast process; a cylindrical neck-like region of varying lengths; a straight and cylindrical trunk with an abdomen of various lengths bearing plumules and elongated egg strings with uniseriate eggs (Kabata, 1979).

The collected specimen closely resembles *P*. *instructa* (Hogans, 1986) and belongs to the group of *Pennella* species with a large (>100 mm) total length, which also includes *P*. *balaenoptera*; *P*. *benzi* Hogans, 2017 and *P*. *filosa*. *Pennella instructa* differs from *P*. *balaenoptera*, *P*. *benzi* and *P*. *filosa* by possession of a cephalothorax anteriorly covered with papillae of apparently even sizes and grouped into four rows with two posterolateral holdfast horns (Hogans, 1986), whereas the others (*P*. *balaenoptera*, *P*. *benzi*, and *P*. *filosa*) possess a cephalothorax covered anteriorly with unorganized papillae of uneven sizes bearing two posterolateral holdfast horns as well as a dorsal horn of different shapes and sizes (Pillai, 1967; Hogans, 2017a).

*Pennella instructa* has previously been reported from *Xiphias gladius* Linnaeus; *Istiophorus platypterus* (Shaw), and *Istiompax indica* (Cuvier) (Wilson, 1917; Pillai, 1967; Hogans, 1986, 2017a). This is the first report of *P*. *instructa* infecting *Seriola lalandi* from a new geographic location i.e. the Atlantic Ocean off South Africa.

***Lernaeenicus*** **Lesueur, 1824**

*Lernaeenicus longiventris* Wilson C.B., 1917

Host: Unknown

Locality: Off South Africa

Material collected and examined: 3♀♀ (broken)

### Re-description:

**Fig. 2:**
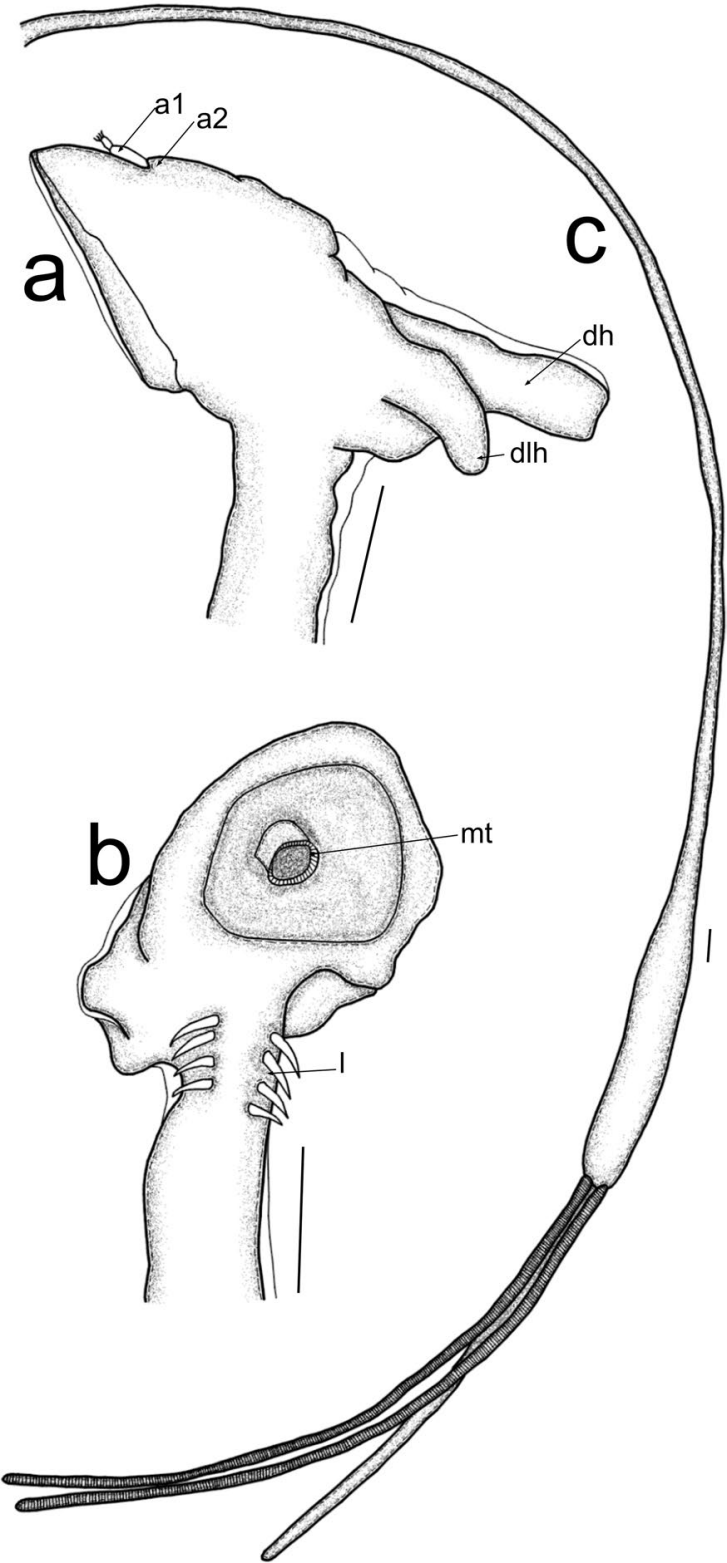
*Lernaeenicus longiventris* Wilson C.B., 1917, post metamorphosed female; a. cephalothorax, lateral view; b. cephalothorax, ventral view; c. habitus without cephalothorax, lateral view. Scale bars a, b = 50 µm; c = 1 mm. (a1 = antennule; a2 = antenna; dh = dorsal holdfast horn; dlh = dorsolateral holdfast horn; mt = mouth tube; l = legs)

Post metamorphosed females [based on three broken specimens, fig. 2]. Body length from tip of neck to tip of abdomen 45.5 mm (33.7-55.7), cephalothorax length 0.8 mm, width 2.1 mm; neck-like region length 25.2 mm (15.4-35 mm), width 0.4 mm; trunk length 6.9 mm (6.3-7.4 mm), width 1.1 mm (1-1.1 mm); abdomen length 13.3 mm (12.1-14.6 mm), width 0.32 mm (0.3-0.4 mm). Anterior part of cephalothorax (fig. 2a) with antennary appendages, antennule (a1) and antenna (a2). The ventral side of cephalothorax (fig. 2c) with mouth tube (mt) and maxillary appendages; the dorsal side (fig. 2a) armed with 3 blunt processes, 1 dorsal horn (dh) and 2 dorsolateral horns (dlh). Neck-like region elongated with four pairs of legs (l) (fig. 2c) anteriorly, expanding into straight, cylindrical trunk, elongated abdomen (ab) and elongated egg strings, with uniseriate eggs.

### Remarks:

*Lernaeenicus* currently has 31 valid species (Walter & Boxshall, 2023) known to infect marine teleosts. Metamorphosed females of this genus are mainly identified by a simple cephalic holdfast with variable lateral, dorsal or both processes of different sizes, a cylindrical neck-like region of different lengths, a straight and cylindrical trunk, with an abdomen of various lengths, and elongated egg strings, with uniseriate eggs (Kabata, 1979).

Specimens collected (figs. 2a-c) closely resemble *Lernaeenicus longiventris* (Knoff & Boeger, 1994) by possession of a cephalothorax with three holdfast horns (2 short dorsolateral and one long dorsal), an elongated neck-like region, a minute trunk 2 times shorter than the abdomen, and an elongated abdomen 2/3 the length of egg strings. There are twelve *Lernaeenicus* species with 3 cephalic horns. *Lernaeenicus longiventris* differs from *L*. *sprattae* (Sowerby, 1806) and *L*. *encrasicoli* (Turton, 1807) by possession of an abdomen and neck longer than the trunk and a dorsal horn longer than than the dorsolateral horns whereas *L*. *sprattae* and *L*. *encrasicoli* possess a minute abdomen, neck shorter than the trunk, longer lateral horns and the dorsal horn as a small protuberance (Kabata, 1979). *Lernaeenicus longiventris* differs from *L*. *kabatai*, *L*. *neglectus* Richiardi, 1877, *L*. *vorax* Richiardi, 1877, *L*. *polynemi* (Bassett-Smith, 1898), *L*. *cerberus* Leigh-Sharpe, 1927 and *L*. *sayori* Yamaguti, 1939 by possession of an abdomen longer than the trunk and a dorsal horn longer than the dorsolateral horns whereas *L*. *kabatai*, *L*. *neglectus*, *L*. *vorax*, *L*. *polynemi*, *L*. *cerberus* and *L*. *sayori* possess an abdomen shorter than the trunk and pronounced cephalic horns of the same lengths (Richiardi, 1877; Leigh-Sharpe, 1927; Pillai, 1967; Oldewage, 1989). *Lernaeenicus longiventris* closely resembles *L*. *hemiramphi* Kirtisinghe, 1932, *L*. *stromatei* Gnanamuthu, 1953 and *L*. *megalepsis* Aneesh, Helna, Kumar & Venmathi Maran, 2021, although it differs from them by possession of short holdfast horns, with the dorsolateral horns shorter than the dorsal horn whereas *L*. *hemiramphi*, *L*. *stromatei*, and *L*. *megalepsis* the cephalic horns are of the same lengths (Gnanamuthu, 1953; Aneesh et al., 2021).

Previous reports of *L*. *longiventris* include infection of the following fish hosts, *Caranx crysos*, *Caranx hippos* (Linnaeus), *Caranx ignobilis* (Forsskål), *Coryphaena hippurus* Linnaeus, *Scomberomorus maculatus* (Mitchill), *Gnathanodon speciosus* (Forsskål), *Mugil cephalus* Linnaeus, *Mugil curema* Valenciennes, *Hyperoglyphe perciformis* (Mitchill), *Urophycis floridana* (Bean & Dresel), *Pogonias cromis* (Linnaeus), *Pomatomus saltatrix* (Linnaeus), *Rachycentron canadum* (Linnaeus), *Sparus aurata* Linnaeus, *Stolephorus commersonnii* Lacepède and *Atherinella brasiliensis* (Quoy & Gaimard) (Wilson, 1917, 1932; Kirtisinghe, 1964; Knoff & Boeger, 1994).

Genus *Lernaeenicus* was inadequately described, with incomplete descriptions of different species (Raja et al., 2016; Ohtsuka et al., 2018). Thus, species of *Lernaeenicus* need a revision (Kabata, 1979) due to discrepancies in the identification e.g. the characteristic feature of a distinct abdomen (Boxshall & Halsey, 2004), while *L*. *quadrilobatus* Yamaguti & Utinomi, 1953 and *L*. *gracilis* (Heller, 1865) lack one. Additionally, amongst the existing species of *Lernaeenicus*, only *L*. *gonostomae* (see Kensley & Grindley, 1973) and *L*. *quadrilobatus* (see Yamaguti & Utinomi, 1953; Kazachenko & Avdeev, 1977) have retained vestiges of a cephalic dorsal shield similar to that in other genera (*Sarcotretes*, *Ophiolernaea* Shiino, 1958, *Creopelates* Shiino, 1958 and *Nagasawanus* Uyeno, 2015). Furthermore, *L*. *quadrilobatus* possesses features characteristic of the genus *Nagasawanus* Uyeno, 2015, such as possession of two pairs of cephalic lobes, mouth tube and maxillary appendages located anteriorly on the ventral surface of cephalothorax, four pairs of legs (first two biramous and last two uniramous) and a minute conical abdomen (Yamaguti & Utinomi, 1953; Kazachenko & Avdeev, 1977; Uyeno, 2015). A complete re-description of *L*. *quadrilobatus* is suggested to validate its taxonomic identity. Species of *Lernaeenicus* either possess cephalic holdfast, thoracic holdfast or both holdfast organs (Kabata, 1979). Species that possess a thoracic holdfast organ (including *L*. *ater* Shiino, 1958, *L. nemipteri* Gnanamuthu, 1953, *L*. *affixus* Wilson C.B., 1917, *L*. *triangularis* Heegaard, 1966, *L*. *polycerus* Wilson C.B., 1917 and *L*. *ramosus* Kirtisinghe, 1956) morphologically resemble each other by presence of a relatively wide abdomen (wider than the width of the neck) but shorter than the trunk and a cephalic attachment plate (see Wilson, 1917; Pillai, 1967). Phylogenetic estimation of some pennellid species (see Fig. 2, Clade III in Yumura et al. (2022)) estimates *L*. *hemirhamphi* basal to a sister grouping of *Pennella* sp. and *L*. *ater*/*L*. *ramosus* sister group. This may be due to *L*. *ater*, *L*. *ramosus* and *Pennella* sp. bearing a thoracic holdfast organ. However, the placement of *Pennella* sp. within the clade of *Lernaeenicus* species also indicates the necessity of a revision of *Lernaeenicus* species.

***Sarcotretes*** **Jungersen, 1911**

*Sarcotretes scopeli* Jungersen, 1911

Host: *Nansenia tenera* Kawaguchi & Butler (Argentiniformes: Microstomatidae)

Locality: Off Namibia (Atlantic Ocean)

Material collected and examined: 6♀♀

Voucher material: 2♀♀ (SAMC-A096498) from *Nansenia tenera* deposited in the Iziko South African Museum, Cape Town, South Africa

### Re-descriptions:

**Fig. 3:**
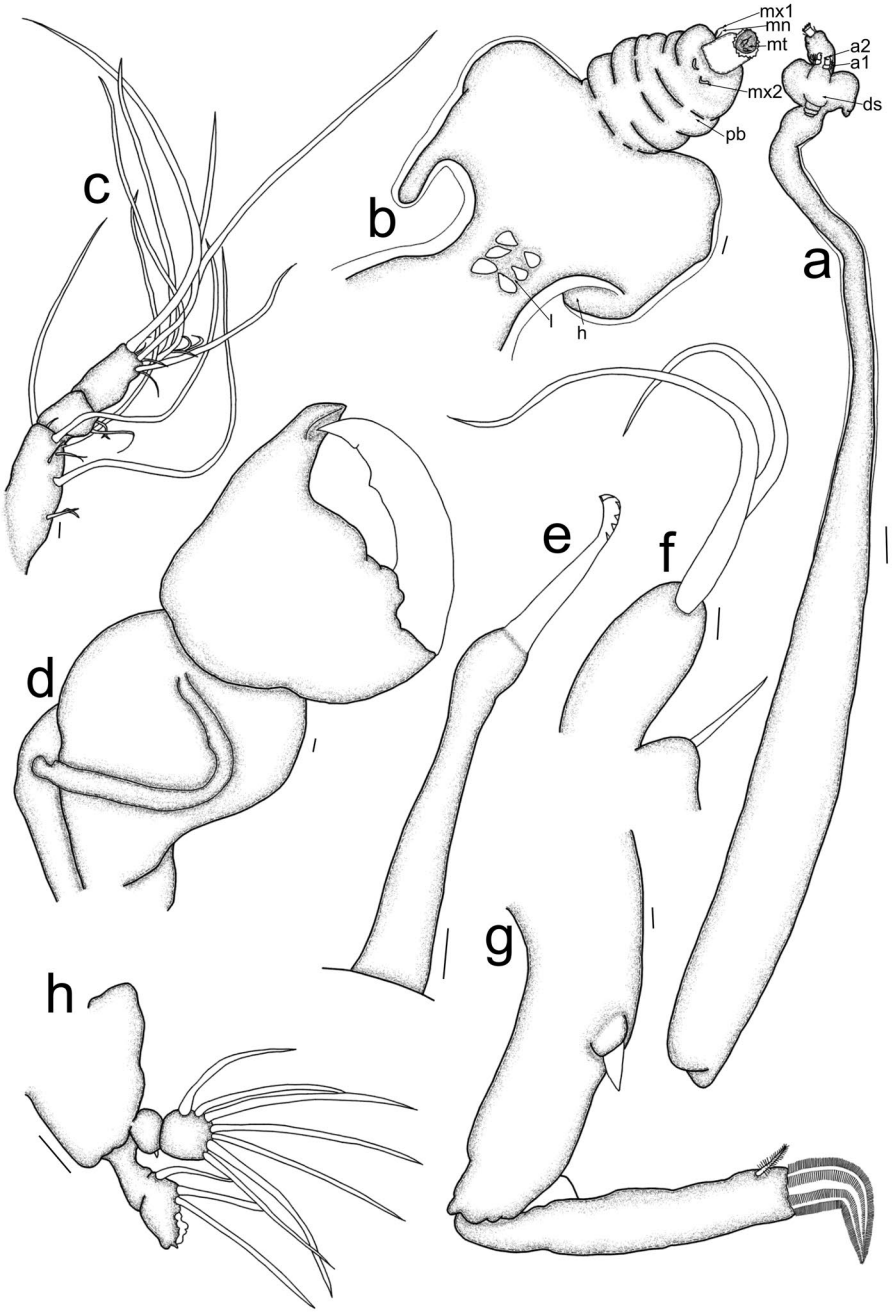
*Sarcotretes scopeli* Jungersen, 1911, post metamorphosed female; a. habitus, cephalothorax dorsal view and trunk lateral view; b. cephalothorax, ventral view; c. antennule; d. antenna; e. mandible; f. maxillule; g. maxilla, h. second leg. Scale bars: a = 1 mm; b, h = 50 µm; c-g = 10 µm. (a1 = antennule; a2 = antenna; ds = dorsal shield rudiment; mt = mouth tube; mn = mandible; mx1 = maxillule; mx2 = maxilla; pb = proboscis; h = holdfast horn; l = legs)

Post metamorphosed females [based on six specimens, fig. 3]. Body length from tip of cephalothorax to the tip of the abdomen 36.7 mm (33.6–38.9 mm), cephalothorax length 2.6 mm (2.1–3.1 mm), width 0.8 mm (0.7–1.0 mm); cephalothorax horn length 1.4 mm (1.0–1.6 mm), width 1.1 mm (0.8–1.4 mm); neck-like region length 14.8 mm (14.0–16.8 mm), width 1.2 mm (0.8–1.3 mm); trunk length 19.4 mm (16.8–21 mm), width 2.5 mm (2.1–2.8 mm); egg-sac length 45.4 mm, width 1.0 mm (0.7–1.1 mm). Cephalothorax (figs. 3a, b) anteriorly soft, longitudinally elongated, with protrusible proboscis (pb), posterior part heavily sclerotized, with lateral cephalothorax horns (h) (figs. 3a, b) extending posteriorly. Cylindrical neck-like region (fig. 3a) with three pairs of legs (l) anteroventrally (fig. 3b). Trunk cylindrical (fig. 3a), with a short abdomen terminally with uniseriated eggs. Antennary appendages (fig. 3a) dorsally on cephalothorax with maxillary appendages anteroventrally on cephalothorax (fig. 3b). Antennule (a1) (fig. 3c) lateral to antenna (a2), 3-segmented, basal segment with 12 setae of different lengths (eight short and four long setae), medial segment with two short setae, terminal segment with 12 apical setae of different lengths (seven short and six long setae). Antenna (fig. 3d) indistinctly 3-4 segmented, first segment robust, with sclerotised ridge; second segment robust, with dentiform process in distal corner accommodating tip of curved claw. Mandible (mn) (fig. 3e) 2-segmented, elongated, with dentiferous margin apically, with about five teeth. Maxillule (mx1) (figs. 3b, 3f) lateral to mouth tube (mt), bilobed, endite with two elongated setae; palp bulbous, with slender apical seta. Maxilla (mx2) (fig. 3g) 2-segmented, lacertus elongated, with stout protuberance medially; brachium slender, with short, plumose clavus at the base of calamus, calamus curved, with several horizontal rows of spiniform setules. Maxilliped absent. Three pairs of legs observed, second leg (fig. 3h) biramous, 2-segmented, endopod first segment armed with a short spine distolaterally, second segment with seven observed setae, exopod first segment unarmed, second segment with only three observed setae.

### Remarks:

Currently *Sarcotretes* has only four species, namely *Sarcotretes eristaliformis* (Brian, 1908); *S*. *scopeli* Jungersen, 1911; *S*. *longirostris* Ho, Nagasawa & Kim I.H., 2007, and *S*. *umitakae* Uyeno, Wakabayashi & Nagasawa, 2012 (Walter & Boxshall, 2023). *Sarcotretes* species are parasitic on teleosts. Post metamorphosed female species of this genus are characterized by a longitudinally elongated cephalothorax with a protrusible proboscis and lateral cephalothorax horns of different lengths extending posteriorly; a cylindrical neck-like region with three pairs of legs (both *S*. *eristaliformis* and *S*. *umitakae* with vestiges of leg four) anteroventrally; cylindrical trunk with a short abdomen terminally and uniseriate eggs (Cherel & Boxshall, 2004; Ho et al., 2007; Uyeno et al., 2012).

*Sarcotretes scopeli* differs from the other *Sarcotretes* species by having a relatively shorter body length (<45 mm) (Hogans, 1988) compared to other *Sarcotretes* species (>45 mm) (Cherel & Boxshall, 2004; Ho et al., 2007; Uyeno et al., 2012). *Sarcotretes scopeli* possesses a neck-like region shorter than the trunk (Hogans, 1988) while *S*. *eristaliformis* possesses a neck-like region of the same length as the trunk (Cherel & Boxshall, 2004) and both *S*. *longirostris* and *S*. *umitakae* possess neck-like regions which is longer than the trunk (Ho et al., 2007; Uyeno et al., 2012).

This is the first report of *S*. *scopeli* infecting *N*. *tenera*, which constitutes a new geographical record off the Namibian coast (Atlantic Ocean). Previously *S*. *scopeli* has been reported from *Benthosema glaciale* (Reinhardt); *Cyclothone atraria* Gilbert; *Diogenichthys atlanticus* (Tåning); *Electrona carlsbergi* (Tåning); *Gonichthys cocco* (Cocco); *Gymnoscopelus nicholsi* (Gilbert); *Gymnoscopelus piabilis* (Whitley); *Hymenogadus gracilis* (Gilbert & Hubbs); *Krefftichthys anderssoni* (Lönnberg); *Lampichthys procerus* (Brauer); *Melanocetus johnsonii* Günther; *Metelectrona ventralis* (Becker); *Symbolophorus evermanni* (Gilbert); *Myctophum punctatum* Rafinesque; *Notoscopelus resplendens* (Richardson); *Polyipnus asteroides* Schultz; *Protomyctophum bolini* (Fraser-Brunner); *Protomyctophum choriodon* Hulley; *Protomyctophum tenisoni* (Norman); *Maurolicus muelleri* (Gmelin); *Scopeloberyx malayanus* (Weber); *Scopeloberyx opisthopterus* (Parr); *Scopeloberyx robustus* (Günther); and *Sternoptyx diaphana* Hermann (Wilson, 1917; Kazachenko & Avdeev, 1977; Boxshall, 1989, 1998; Cherel & Boxshall, 2004).

*Sarcotretes longirostris* Ho, Nagasawa & Kim I.H., 2007

Host: *Centrolophus niger* (Gmelin) (Scombriformes: Centrolophidae)

Locality: off the West coast of South Africa (Atlantic Ocean)

Material collected and examined: 3♀♀ from *Centrolophus niger* and 3♀♀ from unknown hosts off the south coast (Atlantic Ocean, South Africa)

Voucher material: 1♀ (SAMC-A096499) from *Centrolophus niger* deposited in the Iziko South African Museum, Cape Town, South Africa.

### Re-descriptions:

**Fig. 4:**
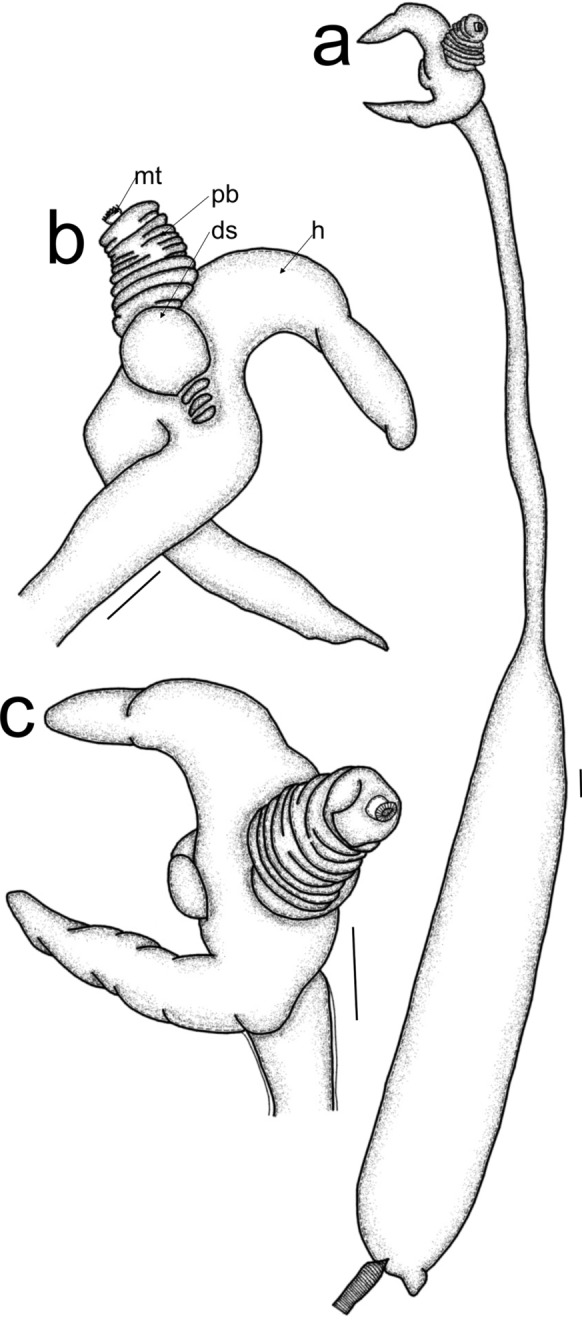
*Sarcotretes longirostris* Ho, Nagasawa & Kim I.H., 2007, post metamorphosed female; a. habitus, cephalothorax anteroventral view and trunk lateral view; b. cephalothorax, posterodorsal view; c. cephalothorax, anteroventral view. Scale bars: a-c = 1 mm. (ds = dorsal shield rudiment; mt = mouth tube; pb = proboscis; h = holdfast horn)

Post metamorphosed females [based on three specimens, fig. 4]. Body length from tip of cephalothorax to tip of abdomen 59.6 mm (48.3–79.8 mm), cephalothorax length 5.6 mm (4.2–8.4 mm), width 1.4 mm (1.12–1.54 mm); cephalothorax horn length 4.9 mm (4.2–5.6 mm), width 1.4 mm; neck-like region length 28.9 mm (19.6–44.8 mm), width 1.0 mm (0.8–1.1 mm); trunk length 21.7 mm (21–22.4 mm), width 3.72 mm (2.8–4.2 mm). Cephalothorax (figs. 4a-c) anteriorly soft, longitudinally elongated, with protrusible proboscis (pb), posterior part heavily sclerotized, with lateral cephalothoracic horns (h) (figs. 4a-c) extending posteriorly. Cylindrical neck-like region (fig. 4a) extending into a cylindrical trunk (fig. 4a), bearing short abdomen terminally with egg strings with uniseriated eggs.

### Remarks:

Material collected (fig. 4) closely resemble those studied in Ho et al. (2007) as *S*. *longirostris*. *Sarcotretes longirostris* differs from both *S*. *eristaliformis* and *S*. *scopeli* by possessing a neck-like region which is longer than the trunk (see fig. 4a), while that of *S*. *eristaliformis* is the same length as the trunk (Cherel & Boxshall, 2004) and that of *S*. *scopeli* is shorter than the trunk (Hogans, 1988). *Sarcotretes longirostris* differs from *S*. *umitakae* by possessing lateral horns (h) of the holdfast of the same width throughout, tapering at tips (Ho et al., 2007) (figs. 4b, c), whereas that of *S*. *umitakae* are proximally bulbous, constrict midway and tapering into slender horns (Uyeno et al., 2012).

This is the first report of *S*. *longirostris* infecting *Centrolophus niger* off the South African coasts. Previously, it has been reported from *Globicephala macrorhynchus* Gray (Ho et al., 2007).

*Sarcotretes gonostomae* (Kensley & Grindley, 1973) **n. comb.**

Syn: *Lernaeenicus gonostomae* Kensley & Grindley, 1973

Host: *Sigmops elongatus* (Günther)

Locality: Off Mozambique (Indian Ocean)

Material examined: 2♀♀ from Iziko South African Museum (Iziko South African Museum voucher numbers A11751 & A13031)

### Re-description:

**Fig. 5:**
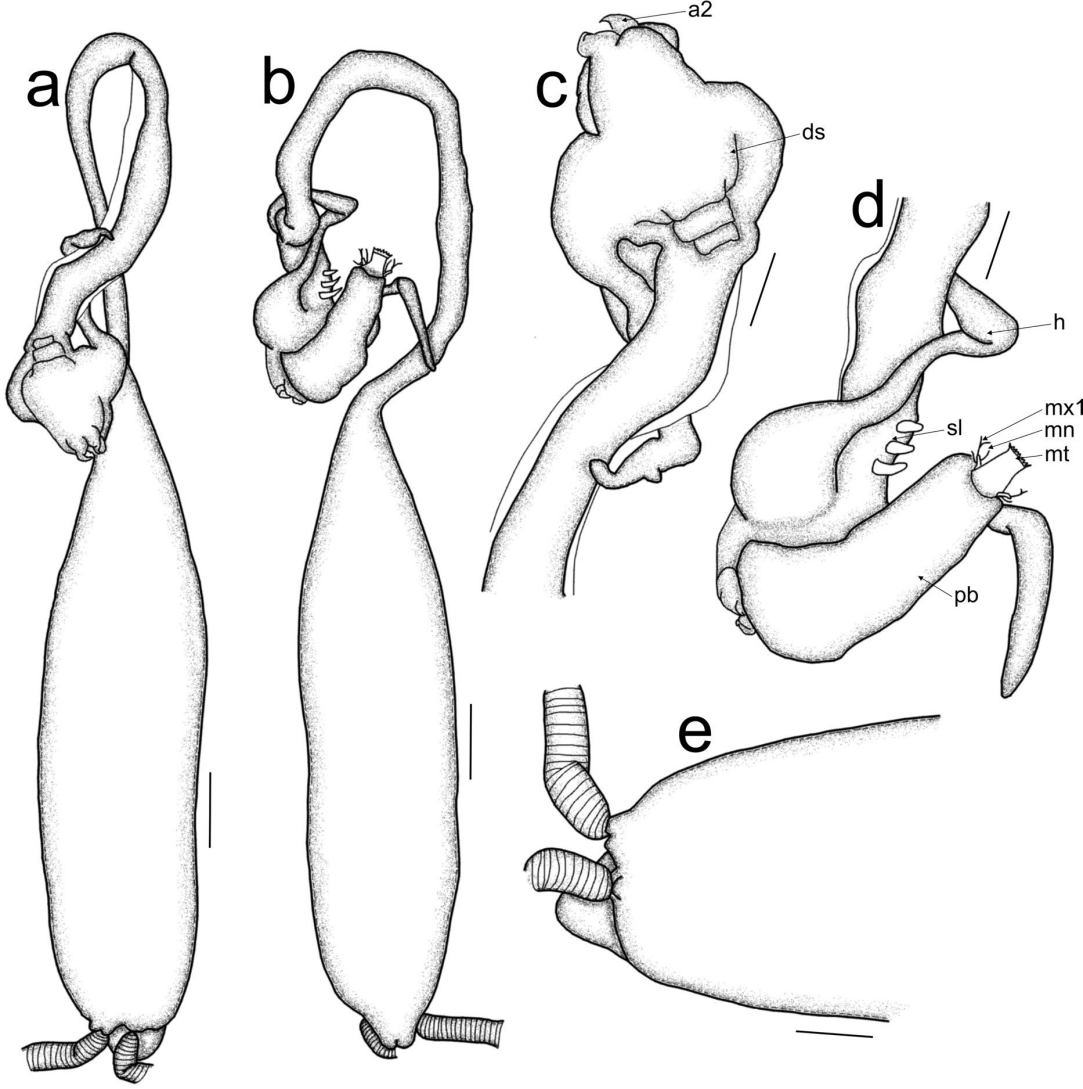
*Sarcotretes gonostomae* (Kensley & Grindley, 1973), post metamorphosed female; a. habitus, cephalothorax dorsal view and trunk ventral view; b. habitus, cephalothorax lateral view and trunk dorsal view; c. cephalothorax, dorsal view; d. cephalothorax, lateral view; e. abdomen, ventral view. Scale bars: a, b = 1 mm; c-e = 50 µm. (a2 = antenna; ds = dorsal shield rudiment; mt = mouth tube; mn = mandible; mx1 = maxillule; mx2 = maxilla; pb = proboscis; h = holdfast horn; l = legs)

Post metamorphosed female [based on one specimen, fig. 5]. Body length from tip of cephalothorax to the tip of the abdomen 26.74 mm, cephalothorax length 4.34 mm, width 1.4 mm; cephalothorax horn length 4.2 mm, width 1.12 mm (1.12 mm bulbous process and 0.14 mm slender process); neck-like region length 12.6 mm, width of 0.56 mm; trunk length 9.8 mm, width 2.1 mm; egg-sac width 0.28 mm. Cephalothorax (figs. 5a-d) anteriorly soft, longitudinally elongated, with protrusible mouth tube (mt), posterior part heavily sclerotized, with lateral holdfast horns (h) (figs. 5a-d), each with a bulbous base and a slender elongation extending posteriorly. Cylindrical neck-like region (figs. 5a-d) with three pairs of legs (l) anteroventrally (fig. 5d), fourth pair not observed. Trunk cylindrical, fusiform (figs. 5a, b), with a short abdomen terminally (figs. 5a, b, e) and egg strings with uniseriated eggs (fig. 5e). Antennary appendages (fig. 5c) dorsally on cephalothorax. Maxillary appendages situated anteroventrally on cephalothorax (fig. 5d), maxillules (mx1) on each side of the mouth tube and maxilla at the base of the proboscis.

### Remarks:

Post metamorphosed females of *Lernaeenicus* as described by Kabata (1979), are characterized by a distinct abdomen of variable length, straight trunk, neck-like region of variable lengths, four thoracic legs and cephalothorax with various holdfast organs made up of processes ranging from only 1 to about 5. *Sarcotretes gonostomae* **n. comb.** differs from *Lernaeenicus* species by possession of a cephalothorax which extends into the elongated proboscis, distinct terga segments on the dorsal side of the cephalothorax, a neck-like region of variable width and a minute abdomen (see figs. 5a-d).

Amongst all Pennellidae genera, the possession of an elongated proboscis occurs only in *Sarcotretes*, *Ophiolernaea* and *Metapeniculus* (Uyeno et al., 2012). *Ophiolernaea* is identified by variable bulbous processes of the holdfast organ on each side of the neck-like region or cephalothorax, four legs and an elongated proboscis, which is longer than the length of the neck-like region and trunk combined (Shiino, 1958; Ho, 1966; Kabata, 1979). The studied specimens possess a proboscis shorter than the neck-like region and trunk. *Metapeniculus* species are characterized by a cephalothorax without a holdfast but with or without anterolateral processes (Castro-Romero & Baeza-Kuroki, 1985) which differs from the current specimens due to the presence of a holdfast. *Sarcotretes* species also possess an elongated mouth cone, but the cephalothorax has lateral holdfast horns extending posteriorly (Wilson, 1917). Thus, the studied specimens belong to *Sarcotretes* with a cephalothorax consisting of two portions, the smooth proboscis-like mouth cone and a heavily sclerotized basal portion, with holdfast organ laterally that extend posteriorly. Additionally, the first two pairs of legs are biramous and leg 3 uniramous, while the neck-like region expands into the elongated trunk with a small, distinct abdomen.

*Sarcotretes gonostomae* **n. comb.** differs from *S*. *scopeli* by possessing an elongated proboscis, elongated holdfast horns, and a neck-like region longer than the trunk, whereas *S*. *scopeli* possesses a relatively short proboscis, short holdfast horns and a neck-like region which is shorter than the trunk. These two species are smaller in total length than the other species. *Sarcotretes gonostomae* **n. comb.** differs from *S*. *eristaliformis* by possessing an elongated oral cone, elongated holdfast horns, and a neck-like region longer than the trunk, whereas *S*. *eristaliformis* possesses a relatively short proboscis, short holdfast horns and a neck-like region of the same length as the trunk (Cherel & Boxshall, 2004). The total length of *S*. *eristaliformis* is about 44.5-58 mm (Cherel & Boxshall, 2004), whilst that of *S*. *gonostomae* **n. comb.** is 26.76 mm. *Sarcotretes gonostomae* **n. comb.** differs from *S*. *longirostris* by possessing holdfast horns with bulbous bases, thinning posteriorly whereas *S. longirostris* has holdfast horns without bulbous bases (Ho et al., 2007). The total length of *S*. *longirostris* is 41.4-74.4 mm (Ho et al., 2007), whereas that of *S*. *gonostomae* **n. comb.** is 26.76 mm. Both *S*. *gonostomae* **n. comb.** and *S*. *longirostris* have elongated holdfast horns and a longer proboscis, but can be distinguished based on the shape of the holdfast horns and the size. *Sarcotretes gonostomae* **n. comb.** closely resembles *S*. *umitakae* with a neck-like region which is longer than the trunk and each holdfast horn with a bulbous base with thin processes extending posteriorly (Uyeno et al., 2012). The total length of *S*. *gonostomae* **n. comb.** is 26.74 mm versus 30.26-50.12 mm in *S*. *umitakae*. The neck-like region is 1.3 times the trunk length in *S*. *gonostomae* **n. comb.** versus 3 times the trunk length in *S*. *umitakae*. The neck-like region of *S*. *umitakae* has a posterior bulge and constriction not observed in *S*. *gonostomae* **n. comb.** *Sarcotretes gonostomae* **n. comb.** has a small abdomen while that of *S*. *umitakae* is minute (Uyeno et al., 2012). Thus, *S*. *gonostomae* **n. comb.** differs from all the existing species of *Sarcotretes*.

***Cardiodectes*** **Wilson C.B., 1917**

*Cardiodectes bellottii* (Richiardi, 1882)

Host: *Lampanyctodes hectoris* (Günther) (Myctophiformes: Myctophidae)

Locality: Off the coast of South Africa

Material collected and examined: 34♀♀

Voucher material: 2♀♀ (SAMC-A096500) deposited in the Iziko South African Museum, Cape Town, South Africa

### Re-description:

**Fig. 6:**
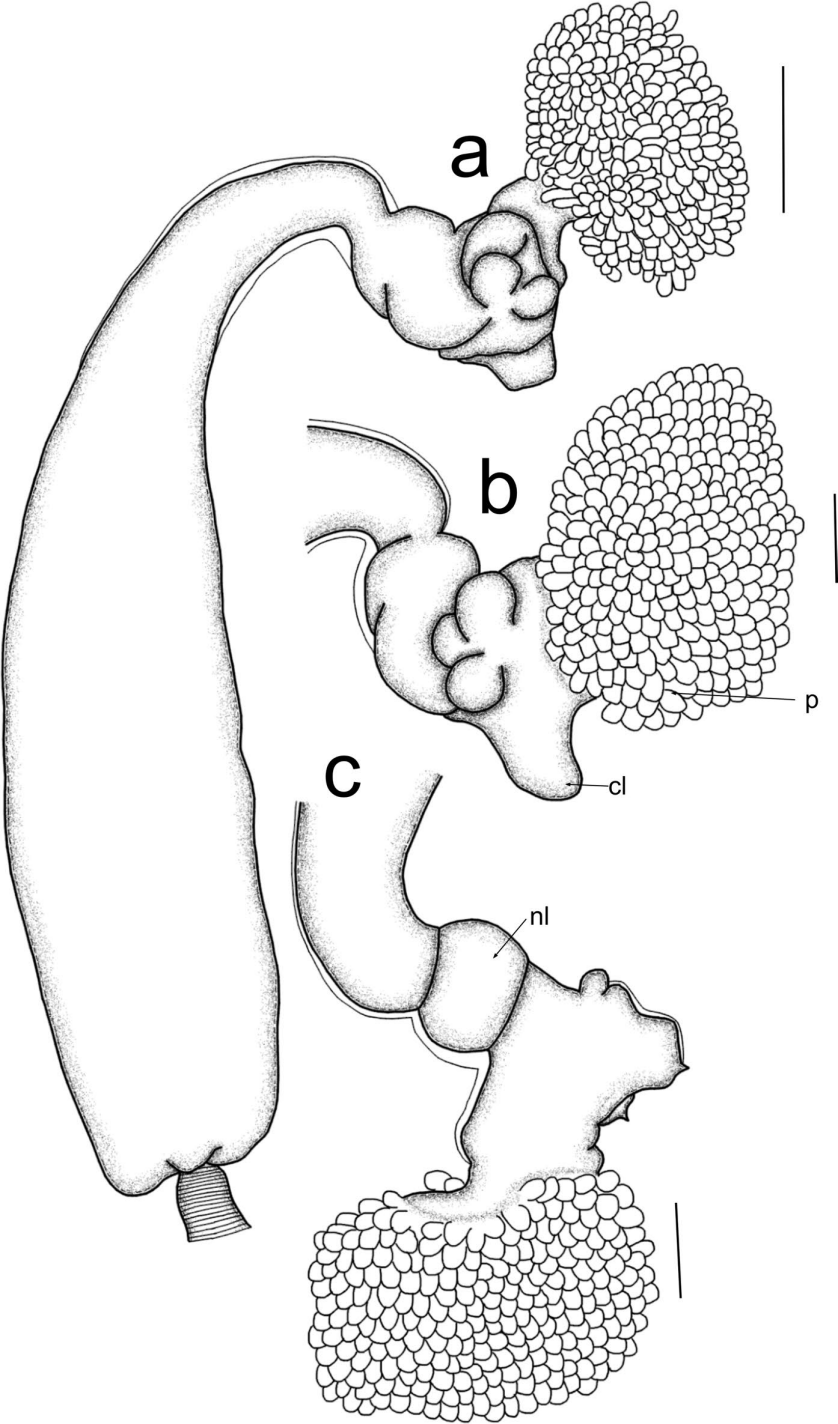
*Cardiodectes bellottii* (Richiardi, 1882), post metamorphosed female; a. habitus, lateral view; b. cephalothorax, anterior view; c. cephalothorax, lateral view. Scale bars: a = 1 mm; b, c = 50 µm. (p = papillae; cl = cephalic lobe; nl = neck lobe)

Post metamorphosed females [based on eight specimens, fig. 6]. Body length from tip of cephalothorax to tip of abdomen 6.8 mm (5.1–7.6 mm), cephalothorax length 1.3 mm (1.1–1.5 mm), width 0.6 mm (0.5-0.7 mm); neck-like region length 1.0 mm (0.8–1.4 mm), width 0.3 mm (0.3-0.4 mm); trunk length 4.3 mm (3.3–5.1 mm), width 1.1 mm (0.9–1.3 mm); abdomen length 0.3 mm (0.2–0.3 mm). Anterior part of cephalothorax (figs. 6a-c) with papillae (p), cephalothorax with simple cephalic lobes (cl) (figs. 6a-c). Neck-like region short, with neck lobe (nl) anteriorly, expanding into a cylindrical trunk, with a minute unilobed abdomen (fig. 6a), and egg strings with uniseriate eggs.

### Remarks:

*Cardiodectes* has 17 valid species (Walter & Boxshall, 2023), infecting marine fishes and with molluscs as intermediate hosts (Ho, 1966; Perkins, 1983; Boxshall, 2000). Females of *Cardiodectes* are mainly characterized by a cephalothorax with branched papillae anteriorly (figs. 6a-c), with or without cephalic lobes, a neck-like region of variable lengths, a cylindrical trunk of variable length and width, with or without a distinct abdomen, and straight or spiral egg strings with uniseriate eggs. Adult females are divided into two groups, i.e. *medusaeus* (including *Cardiodectes bellottii* (Richiardi, 1882); *C*. *anchorellae* Brian & Gray, 1928; *C*. *frondosus* Schuurmans Stekhoven J.H. Jr, 1937; *C*. *cristatus* Shiino, 1958, and *C*. *longicervicus* Shiino, 1958) and *rubosus* (including *C*. *rubosus* Leigh-Sharpe, 1934; *C*. *hardenbergi* Markevich, 1936; *C*. *krishnai* Sebastian, 1968; *C*. *rotundicaudatus* Izawa, 1970; *C*. *boxshalli* Bellwood, 1981; *C*. *spiralis* Bellwood, 1981; *C*. *asper* Uyeno & Nagasawa, 2010; *C*. *bertrandi* Uyeno & Nagasawa, 2010; *C*. *bellwoodi* Uyeno, 2013; *C*. *shini* Uyeno, 2013; *C*. *roatanensis* Suárez-Morales, Vásquez-Yeomans & Vidotto, 2022, and *C*. *vampire* Aneesh, Helna, Kumar & Venmathi Maran, 2023). Members of the *medusaeus* group are known for possession of a distinct abdomen whereas those of the *rubosus* group lack a distinct abdomen.

**Fig. 7:**
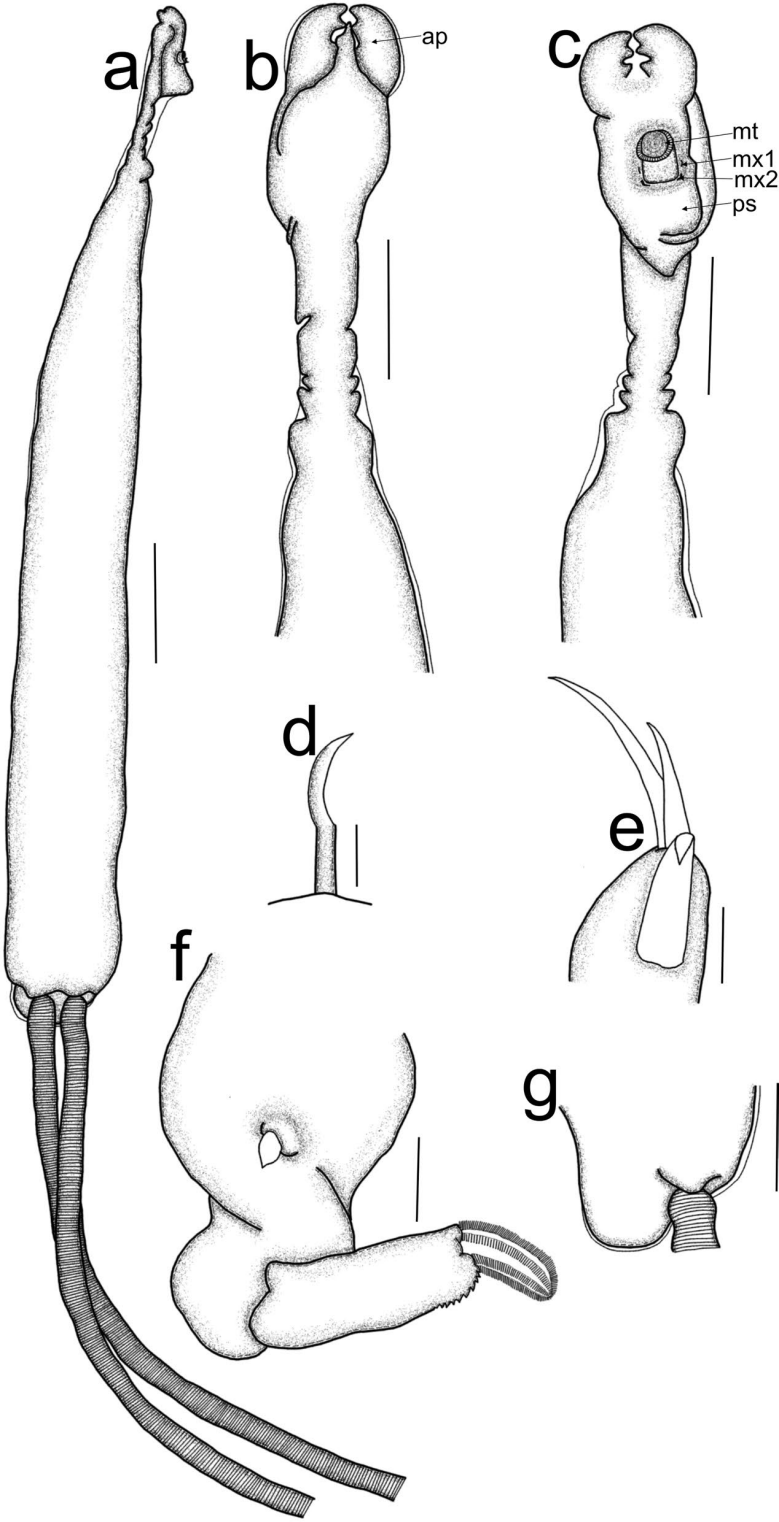
*Propeniculus stromatei* (Gnanamuthu, 1951), post metamorphosed female; a. habitus, cephalothorax lateral view and trunk ventral view; b. cephalothorax, dorsal view; c. cephalothorax, ventral view; d. mandible; e. maxillule; f. maxilla; g. abdomen, lateral view. Scale bars: a = 1 mm; b, c, g = 50 µm; d = 5 µm; e, f = 10 µm. (ap = antennary process; mt = mouth tube; mx1 = maxillule; mx2 = maxilla; ps = posterior swelling)

Material collected (figs. 6a-c) closely resembles those studied by Shiino (1958), Boxshall (2000) and Hogans (2017b) as *C*. *bellottii*. *Cardiodectes bellottii* differs from *C*. *anchorellae* by possessing a unilobed abdomen (fig. 6a) whereas that of *C*. *anchorellae* is bilobed (Pillai, 1967) and from *C*. *longicervicus* by possessing a neck-like region which is shorter than the trunk (fig. 6a) whereas that of *C*. *longicervicus* is the same length as the trunk (Shiino, 1958). Additionally, *C*. *bellottii* differs from *C*. *cristatus* and *C*. *frondosus* by possessing a short abdomen (fig. 6a) whereas those of *C*. *cristatus* (see Abb 3 in Shiino (1958)) and *C*. *frondosus* (see FIGS 1 and 7 in Schuurmans Stekhoven (1937)) are elongated.

***Propeniculus*** Castro-Romero, 2014

*Propeniculus stromatei* (Gnanamuthu, 1951)

Host: *Pomadasys commersonnii* (Lacepède) (Eupercaria: Haemulidae)

Material collected and examined: 3♀♀ from *Pomadasys commersonnii* from the Indian Ocean, South Africa and 1♀ from *Rhabdosargus holubi* (Steindachner, 1881) (Eupercaria: Sparidae) from the Indian Ocean, South Africa

Voucher material: 1♀ (SAMC-A096501) from *Pomadasys commersonnii* deposited in the Iziko South African Museum, Cape Town, South Africa

### Re-description:

Post metamorphosed females [based on four specimens, fig. 7]. Body length from tip of cephalothorax to tip of abdomen 9.5 mm (9.1–9.8 mm), cephalothorax length 0.7 mm (0.7–0.8 mm), width 0.4 mm (0.4 mm); neck-like region length 1.0 mm (0.8–1.1 mm), width 0.1 mm (0.1 mm); fourth segment length 0.14 mm (0.14 mm), width 0.28 mm (0.28 mm); trunk length 8.1 mm (7.6–8.8 mm), width 1.2 mm (1.0–1.4 mm); abdomen length 0.3 mm (0.2–0.3 mm); egg-sac length 9.8 mm, width 1.0 mm.

The tip of the cephalothorax (figs. 7a-c) bifid, dorsal side (fig. 7b) sclerotized, with raised surface, ventral surface (fig. 7c) sclerotized anteriorly, posteriorly soft, with swelling (ps). Neck-like region cylindrical (fig. 7a), divided into four somites (figs. 7a-c) (each somite with a pair of legs). Trunk (fig. 7a) elongated and cylindrical, with visible abdomen (figs. 7a, g). Egg strings (fig. 7a) elongate, with uniseriate eggs.

Antennary appendages not observed. Maxillary appendages situated on the ventral side of the cephalothorax (fig. 7c). Mouth tube (mt) protrusible (fig. 7c). Mandible (mn) (fig. 7d) with curving tip without noticeable teeth. Maxillule (mx1) (fig. 7e) situated posterolateral to the mouth tube, 1-segmented, endite with two apical setae of different lengths, palp with single seta. Maxilla (mx2) (fig. 7f) 2-segmented, lacertus robust, with stout protuberance medially; brachium slender, bearing denticles on the posterodistal margin, calamus claw-like, with rows of spiniform setules. Maxilliped absent. Rudiments of four pairs of legs observed. Abdomen (fig. 7g) slightly elongated.

### Remarks:

According to Castro-Romero (2014), *Propeniculus* species closely resemble *Peniculus* species, but possess features primitive compared to *Peniculus* species, including aspects of the cephalothorax (e.g. buccal structure and posterior swelling), a reinforced neck-like region with somites (with exception to *Peniculus communis* Leigh-Sharpe, 1934) and the attachment of egg strings to the abdomen aided by several flanges. *Propeniculus* species are mainly characterized by a simple cephalothorax, a short segmented neck-like region with four segments of different sizes, a straight trunk with a simple abdomen (Castro-Romero, 2014). *Propeniculus* consists of five species, namely *Propeniculus sciaenae* (Gnanamuthu, 1951); *P*. *scomberi* (Gnanamuthu, 1951); *P*. *stromatei* (Gnanamuthu, 1951); *P*. *theraponi* (Gnanamuthu, 1951); and *P*. *trichiuri* (Gnanamuthu, 1951) (Walter & Boxshall, 2023).

Females of *Propeniculus* species can be differentiated from one another by the length of the cephalothorax relative to the neck-like region and trunk lengths, the cephalothorax shape, the length of the neck-like region and the width of the fourth somite in relation to the trunk width and the length of the abdomen (Gnanamuthu, 1951, 1952; Castro-Romero, 2014).

*Propeniculus stromatei* differs from *P*. *sciaenae* and *P*. *theraponi* by possession of a prominent posterior swelling, with a short, protrusible mouth tube whereas *P*. *sciaenae* and *P*. *theraponi* possess a less prominent posterior swelling, but larger mouth cone (see Figs. 12 and 14 in Gnanamuthu (1951)). *Propeniculus stromatei* differs from *P*. *scomberi* in the structure of the cephalothorax, with *P*. *stromatei* with a symmetrical cephalothorax with a posterior swelling and even anteroventral surface whereas the cephalothorax of *P*. *scomberi* is asymmetrical, without a posterior swelling, but with a swollen ventral surface (see Figs. 2a-g in Gnanamuthu (1952)). *Propeniculus stromatei* resembles *P*. *trichiuri*. However, the cephalothorax of *P*. *stromatei* has a less protruding posterior swelling ventrally and is flattened dorsally, with a neck-like region longer than the cephalothorax (Gnanamuthu, 1952) whereas *P*. *trichiuri* (see Figs. 1 and 4 in Gnanamuthu (1951)) possesses a more protruding posterior swelling on the ventral side of the cephalothorax as well as a protruding dorsal side and has a neck-like region shorter than the cephalothorax. Additionally, the trunk of *P*. *stromatei* is approximately eight times longer than the neck-like region (Gnanamuthu, 1952) whereas that of *P*. *trichiuri* is more than eleven times the neck-like region (Fig. 1 in Gnanamuthu, 1951).

Previously, *P*. *stromatei* has been reported from *Parastromateus niger* (Bloch) (Gnanamuthu, 1952). Both *P. niger* and *Pomadasys commersonii* are reef-associated while *Rhabdosargus holubi* is demersal (Froese & Pauly, 2023). Other *Propeniculus* species were reported from several different fish species, namely *P*. *sciaenae* from *Daysciaena albida* (Cuvier), *P*. *scomberi* from *Rastrelliger kanagurta* (Cuvier), *P*. *theraponi* from *Terapon jarbua* (Forsskål) and *P*. *trichiuri* from *Trichiurus lepturus* Linnaeus, *Lepturacanthus savala* (Cuvier), and *Eupleurogrammus muticus* (Gray) (Gnanamuthu, 1951, 1952; Castro-Romero, 2014).

## Phylogenetic analysis

### Results and discussion:

The complete dataset consists of 54 sequences including nine genera belonging to Pennellidae and two Caligidae species as outgroup taxa. The sequences are 507-683 base pairs long except that of *S*. *scopeli* which is only 365 base pairs long. Even though the COI gene is not ideal for estimating relationships among genera it still provides an hypothesis of relationships to be tested using more suitable slower-evolving genes.

**Fig. 8:**
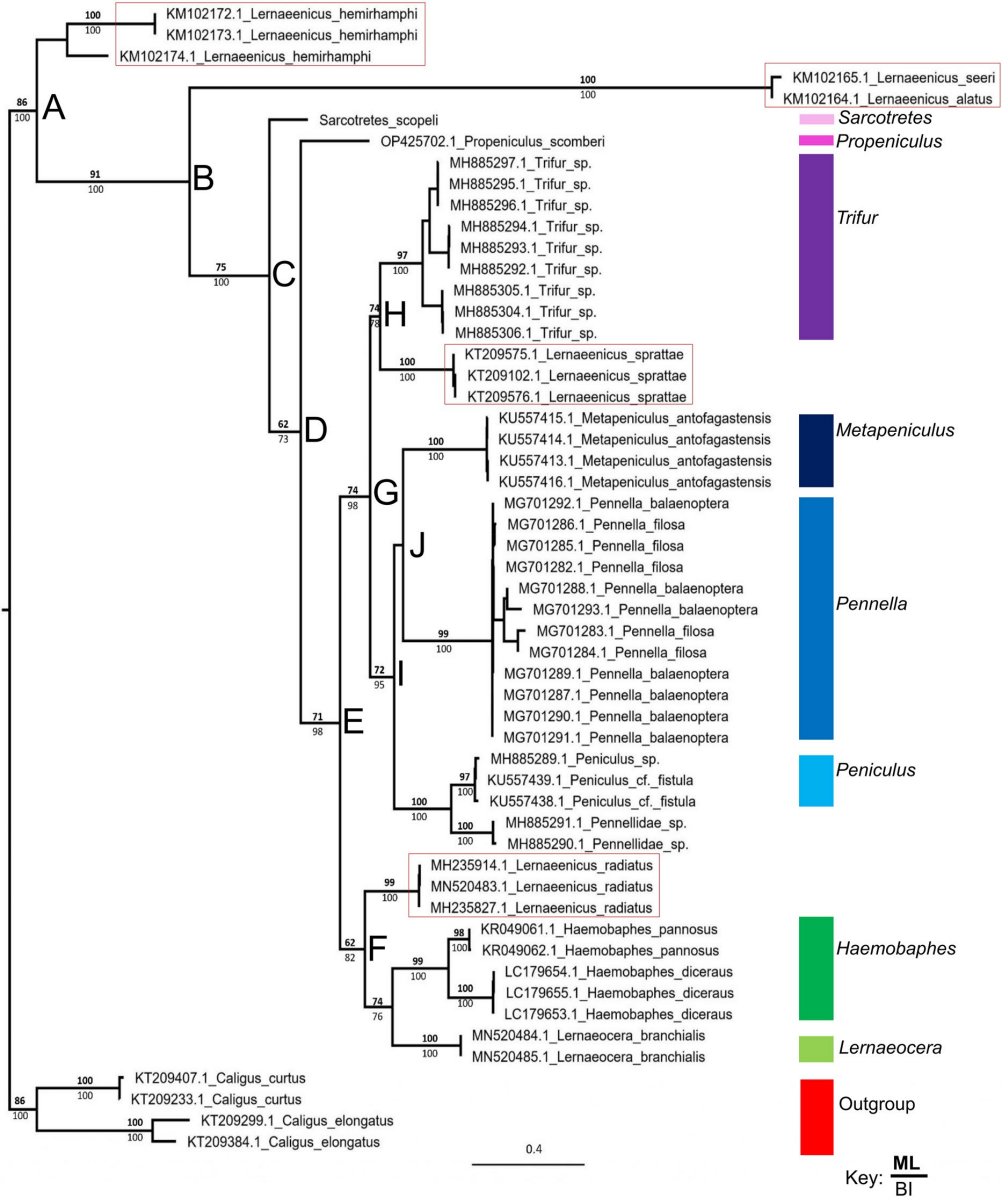
The estimated phylogram of some Pennellidae genera with representatives of *Caligus* as outgroup taxa, using maximum likelihood and Bayesian inference of the partial COI (cytochrome oxidase I) gene. Values above the lines indicate bootstrap support (%) of the ML analysis while those below the lines indicate the posterior probabilities (%) of the Bayesian analysis.

Both the maximum likelihood and Bayesian analyses of the COI dataset estimated Pennellidae as a highly supported monophyletic group (fig. 8). Even though most genera included in the dataset with more than one sequence (*Trifur*, *Metapeniculus*, *Pennella*, *Peniculus*, *Haemobaphes* and *Lernaeocera*) form highly supported monophyletic groups, it may be due to most sequences possibly being conspecific (except for those of *Pennella* and *Haemobaphes*). *Sarcotretes scopeli* (although a much shorter sequence of only 365 bp) is basal to all other genera from node D supporting the estimated morphological phylogeny in Boxshall (1986) (see Fig. 5 in Boxshall (1986)). According to Castro-Romero (2014), the structure of the mouth tube of *Propeniculus* is primitive to all pennellids which attach to the host fins, including *Peniculus*, *Metapeniculus* and *Peniculisa*. In the estimated phylogram (fig. 8) *Propeniculus* is estimated as the basal group to all the remaining genera and not only to *Peniculus* and *Metapeniculus*, while *Metapeniculus* and *Pennella* form an unsupported sister grouping (node J) with *Peniculus* (and Pennellidae species) basal to this grouping (node I). Further support for the previous morphological estimation (specifically regarding the possession of a sigmoid trunk) (Boxshall, 1986) is the well supported sister grouping of *Haemobaphes* and *Lernaeocera*. Notably in the estimated phylogeny (fig. 8) is the species of *Lernaeenicus* which are polyphyletic with a well-supported clade formed by *L*. *seeri* Kirtisinghe, 1934 and *L*. *alatus* Rangnekar, 1962 basal to all included genera (node B), *L*. *sprattae* (Sowerby, 1806) as a sister group of *Trifur* sp. (node H) and *L*. *radiatus* as a sister group of the *Haemobaphes*/*Lernaeocera* sister group (node F). Thus, it seems that some *Lernaeenicus* species were misidentified or rather that *Lernaeenicus* is in serious need of revision. According to Boxshall (1986), *Lernaeenicus* can be distinguished by their possession of an elongated body which is similar to other Pennellidae genera including *Sarcotretes*, *Peniculus*, *Propeniculus*, *Pennella*, *Exopenna* Boxshall, 1986, *Metapeniculus*, *Pseudopeniculus* and *Peniculisa*. Furthermore, distinguishing features mentioned by Kabata (1979) include many possible variations which are also shared by other genera. Regarding the estimated relationships in this study, *L*. *sprattae* and *Trifur* sp. share morphological similarities including the possession of three holdfast horns on the cephalothorax (2 lateral and 1 dorsal) and the possession of an abdomen shorter than the trunk. Furthermore, *Lernaeenicus radiatus*, *Lernaeocera branchialis* (Linnaeus, 1767), *Haemobaphes pannosus* Kabata, 1979 and *H*. *diacerus* Wilson C.B., 1917 share the possession of holdfast organs situated in a horizontal plane at the base of the cephalothorax, posterior holdfast organ (on neck-like region) and an abdomen shorter than the trunk. Therefore, even though more data is needed for a more conclusive estimation of the relationships amongst the genera of Pennellidae, it is clear that specifically *Lernaeenicus* is in need of thorough revision of all current species.

## Conclusions

New geographical records are reported for *Pennella instructa*, *Propeniculus stromatei*, *S*. *scopeli*, *S*. *longirostris*, and *L*. *longiventris* from southern African waters with new host records for *P*. *instructa* on *Seriola lalandi*, *P*. *stromatei* on *Rhabdosargus holubi* and *Pomadasys commersonnii*, *S*. *scopeli* on *Nansenia tenera* and *S*. *longirostris* on *Centrolophus niger*. Re-descriptions are provided for *P*. *stromatei* and *S*. *scopeli* and *S*. *gonostomae* **n. comb.** (synonymy *L*. *gonostomae*) from Iziko South African Museum. Additionally, an estimation of the phylogenetic relationships amongst some of the Pennellidae genera was attempted using COI sequences available on Genbank with the addition of a generated sequence for *S*. *scopeli*. This estimation emphasizes the need of revising the species of *Lernaeenicus* specifically due to their polyphyletic grouping in the phylogram.

## Data Availability

Voucher specimens were deposited into the Iziko South African Museum, Cape Town, South Africa.

## References

[CR1] Aneesh PT, Helna AK, Kumar AB, Venmathi Maran BA (2021). A new species of parasitic copepod of the genus *Lernaeenicus* Lesueur, 1824 (Siphonostomatoida: Pennellidae) from the torpedo scad *Megalaspis cordyla* (Linnaeus) off Kerala coast of Arabian Sea, India. Marine Biology Research.

[CR2] Barnard KH (1955). South African Parasitic Copepoda. Annals of the South African Museum.

[CR3] Boxshall GA (1986). A new genus and two new species of Pennellidae (Copepoda: Siphonostomatoida) and an analysis of evolution within the family. Systematic Parasitology.

[CR4] Boxshall GA (1989). Parasitic copepods of fishes: A new genus of the Hatschekiidae from New Caledonia, and new records of the Pennellidae, Sphyriidae and Lernanthropidae from the South Atlantic and South Pacific. Systematic Parasitology.

[CR5] Boxshall GA (1998). Host specificity in copepod parasites of deep-sea fishes. Journal of Marine Systems.

[CR6] Boxshall GA (2000). Parasitic copepods (Copepoda: Siphonostomatoida) from deep-sea and mid-water fishes. Systematic Parasitology.

[CR7] Boxshall GA, Halsey SH (2004). An Introduction to Copepod Diversity.

[CR8] Castro-Romero R (2014). Two new genera of pennellids (Copepoda, Siphonostomatoida): *Propeniculus* and *Pseudopeniculus*, each with a new combination, *Propeniculus trichiuri* (Gnanamuthu, 1951) and *Pseudopeniculus asinus* (Kabata & Wilkes, 1977). Crustaceana.

[CR9] Castro-Romero R, Baeza-Kuroki H (1985). *Metapeniculus antofagastensis* gen. et sp. nov. (Copepoda, Pennellidae) parasitic on two inshore fishes of Antofagasta, Chile. South Pacific. Crustaceana.

[CR10] Cherel Y, Boxshall GA (2004). *Sarcotretes* (Copepoda: Pennellidae) parasitizing myctophid fishes in the Southern Ocean: new information from seabird diet. Journal of Parasitology.

[CR11] Darriba D, Taboada GL, Doallo R, Posada D (2012). JModelTest 2: More Models, new heuristics and parallel computing. Nature Methods.

[CR12] Dippenaar SM (2004). Reported siphonostomatoid copepods parasitic on marine fishes of Southern Africa. Crustaceana.

[CR13] Froese, R., & Pauly, D. Editors. (2023). FishBase. World Wide Web electronic publication. http://www.fishbase.org version (04/2023). Accessed on 06 April 2023.

[CR14] Geller J, Meyer C, Parker M, Hawk H (2013). Redesign of PCR primers for mitochondrial cytochrome c oxidase subunit I for marine invertebrates and application in all-taxa biotic surveys. Molecular Ecological Resources.

[CR15] Gnanamuthu CP (1951). Three new species of lernaeid copepods parasitic on South Indian fish. Annals and Magazine of Natural History.

[CR16] Gnanamuthu CP (1952). Two new species of copepods of the genus *Peniculus* parasitic on Madras fishes. Records of the Indian Museum, Calcutta.

[CR17] Gnanamuthu CP (1953). Three lernaeid copepods parasitic on South Indian fishes. Journal of Parasitology.

[CR18] Ho JS (1966). Three species of Formosan copepods parasitic on fishes. Crustaceana.

[CR19] Ho JS, Nagasawa K, Kim IH (2007). *Sarcotretes longirostris* n. sp. (Copepoda, Pennellidae) parasitic on bluefin driftfish (*Psenes pellucidus*) found in the stomach of the short-finned pilot whale caught off Japan. Journal of Crustacean Biology.

[CR20] Hogans WE (1986). Redescription of *Pennella instructa* Wilson, 1917 (Copepoda: Pennellidae) from the swordfish (*Xiphias gladius L*.). Canadian Journal of Zoology.

[CR21] Hogans WE (1988). Review of *Sarcotretes* (Copepoda: Pennellidae) from midwater and demersal fishes in the North Atlantic Ocean. Canadian Journal of Zoology.

[CR22] Hogans WE (2017). Review of *Pennella* Oken, 1816 (Copepoda: Pennellidae) with a description of *Pennella benzi* sp nov., a parasite of Escolar, *Lepidocybium flavobrunneum* (Pisces) in the northwest Atlantic Ocean. Zootaxa.

[CR23] Hogans WE (2017). *ardiodectes medusaeus* (Copepoda: Pennellidae) a synonym of *Cardiodectes bellottii* , a parasite of mid-water fishes in the North Atlantic Ocean and Mediterranean Sea. Proceedings of the Biological Society of Washington.

[CR24] Kabata Z (1979). Parasitic Copepoda of British fishes.

[CR25] Kazachenko, V.N., & Avdeev, G.V. (1977). Paraziticheskie kopepody (Copepoda, Crustacea) v sborakh 57-go reisa NIS 'Vityaz' v zapadnoi tropicheskoi chasti Tikhogo okeana i moryakh Indomalaiskogo arkhipelaga. Parasitic copepods (Crustacea) collected during the 57th cruise of the RV 'Vityaz' in the western tropical Pacific and seas of the Indo-malayan Archipelago. *Glubokovodnye biologicheskie issledovaniya v zapadnoi tropicheskoi chasti Tikhogo okeana. Trudy Instituta Okeanologii*, 107, 30–48. (Russian with English summary).

[CR26] Kensley B, Grindley JR (1973). South African parasitic Copepoda. Annals of the South African Museum.

[CR27] Kirtisinghe P (1964). A review of the parasitic copepods of fish recorded from Ceylon, with descriptions of additional forms. Bulletin of the Fisheries Research Station.

[CR28] Knoff M, Boeger WA (1994). Expanded description of the female of *Lernaeenicus longiventris* Wilson, 1917, (Copepoda, Siphonostomatoida, Pennellidae) based on specimens from *Mugil platanus* Günter, (Perciformes, Mugilidae) of the State of Rio de Janeiro, Brazil. Memórias do Instituto Oswaldo Cruz.

[CR29] Leigh-Sharpe WH (1927). Report on a parasitic copepod of *Atherina pinguis* (*Lernaeenicus cerberus* sp. n.). Zoological results of the Cambridge Expedition to the Suez Canal, 1924, no. X. Transactions of the Zoological Society of London.

[CR30] Leray M, Yang JY, Meyer CP, Mills SC, Agudelo N, Ranwez V, Boehm JT, Machida RJ (2013). A new versatile primer set targeting a short fragment of the mitochondrial COI region for metabarcoding metazoan diversity: Application for characterizing coral reef fish gut contents. Frontiers in Zoology.

[CR31] Maddison DR, Maddison WP (2001). MacClade version 4: analysis of phylogeny and character evolution.

[CR32] Nguyen LT, Schmidt HA, von Haeseler A, Minh BQ (2015). IQ-TREE: A fast and effective stochastic algorithm for estimating maximum likelihood phylogenies. Molecular Biology and Evolution.

[CR33] Ohtsuka S, Lindsay DJ, Izawa K (2018). A new genus and species of the family Pennellidae (Copepoda, Siphonostomatoida) infecting the Pacific viperfish *Chauliodus macouni*. Parasite.

[CR34] Oldewage WH (1989). A new species of *Lernaeenicus* (Copepoda: Siphonostomatoida) from southern Africa. South African Journal of Zoology.

[CR35] Osuna-Cabanillas JM, Morales-Serna FN, Venmathi Maran BA, Cruz-Barraza JA (2023). Redescription of *Lernaeenicus longiventris* Wilson, 1917 (Copepoda: Pennellidae) parasitic on the Pacific Crevalle Jack *Caranx caninus* (Carangidae) through morphological and molecular analyses. Acta Parasitologica.

[CR36] Perkins PS (1983). The life history of *Cardiodectes medusaeus* (Wilson), a copepod parasite of lanternfishes (Myctophidae). Journal of Crustacean Biology.

[CR37] Pillai, N.K. (1967). Copepods parasitic on Indian marine fishes. A review. In: Proceedings of the Symposium on Crustacea. *Symposium Series, Marine Biological Association of India*, 5, 1556–1680.

[CR38] Raja K, Saravanakumar A, Gopalakrishnan A, Vijayakumar R, Hwang UW, Maran BA (2016). The genus *Lernaeenicus* Lesueur (Copepoda, Siphonostomatoida, Pennellidae) in India: a checklist with notes on its taxonomy and ecology. Zootaxa.

[CR39] Rambaut, A. (2016). Figtree v1.4.3: Tree Figure Drawing Tool.

[CR40] Richiardi, S. (1877). Descrizioni di due specie nuove di *Lernaeenicus* Les. con osservazioni intorno a questo ed al generi *Lernaeocera* Bl. e *Lernaeonema* M. Edw. *Atti della Società Toscana di Scienze Naturali, Processi Verbali e Memorie, Serie B*, 3, 195–206.

[CR41] Ronquist F, Teslenko M, Van Der Mark P, Ayres DL, Darling A, Hohna S, Larget B, Liu L, Suchard MA, Huelsenbeck JP (2012). MrBayes 3.2: Efficient bayesian phylogenetic inference and model choice across a large model space. Systematic Biology.

[CR42] Schuurmans Stekhoven JH (1937). II. Crustacea Parasitica. I. Parasitica Copepoda. Résultats Scientifiques des Croissières du Navire-École 'Mercator', 1. Mémoires du Musée Royal d'Histoire Naturelle de Belgique.

[CR43] Shiino SM (1958). Copepods parasitic on Japanese fishes. 17. Lerneidae. Reports of the Faculty of Fisheries, Prefectural University of Mie.

[CR44] Thompson JD, Gibson TJ, Plewniak F, Jeanmougin F, Higgins DG (1997). The CLUSTAL_X windows interface: fexible strategies for multiple sequence alignment aided by quality analysis tools. Nucleic Acids Research.

[CR45] Uyeno D (2015). Systematic revision of the pennellid genus *Creopelates* Shiino, 1958 (Copepoda: Siphonostomatoida) and the proposal of a new genus. Zootaxa.

[CR46] Uyeno D, Wakabayashi K, Nagasawa K (2012). A new species of parasitic copepod, *Sarcotretes umitakae* sp. n. (Siphonostomatoida, Pennellidae), on the rattail (Actinopterygii, Macrouridae) from the East China Sea. Japan. Zookeys.

[CR47] Walter, T.C., & Boxshall, G. (2023). World of Copepods Database. Pennellidae Burmeister, 1835. https://www.marinespecies.org/aphia.php?p=taxdetails&id=135532. Accessed on 11 November 2023.

[CR48] Wilson CB (1917). North American parasitic copepods belonging to the family Lernaeidae with a revision of the entire family. Proceedings of the United States National Museum.

[CR49] Wilson CB (1932). The copepods of the Woods Hole region, Massachusetts. Bulletin of the United States National Museum.

[CR50] Yamaguti S, Utinomi H (1953). *Lernaeenicus quadrilobatus* n. sp. (Copepoda, Lernaeidae) parasitic on the lantern-fish *Diaphus coeruleus*. Publications of the Seto Marine Biological Laboratory.

[CR51] Yumura N, Adachi K, Nitta M, Kondo Y, Komeda S, Wakabayashi K, Fukuchi J, Boxshall GA, Ohtsuka S (2022). Exploring evolutionary trends within the Pennellidae (Copepoda: Siphonostomatoida) using molecular data. Systematic Parasitology.

[CR52] Yumura N, Nishikawa J, Ohtsuka S (2024). A New Species and a New Genus of the Family Pennellidae (Copepoda: Siphonostomatoida) Parasitic on North Pacific Lightfish Maurolicus japonicus (Actinopterygii. Stomiiformes: Sternoptychidae) Collected from Suruga Bay Japan. Species Diversity.

